# Clinical guidelines for the diagnosis and treatment of vitreoretinal lymphoma in Chinese patients (2024)

**DOI:** 10.1186/s40662-025-00434-4

**Published:** 2025-05-26

**Authors:** Xiao Zhang, Xiaoyan Peng, Dan Liang, Yaolong Chen, Wei Zhang, Yan Zhang, Meifen Zhang, Peizeng Yang, the Ocular Immunology Group of the Chinese Medical Association Ophthalmology Branch, Xiao Zhang, Xiaoyan Peng, Dan Liang, Wei Zhang, Yan Zhang, Meifen Zhang, Peizeng Yang, Weidong Cheng, Fei Gao, Jin Geng, Wubi Li, Zhanwu Lin, Yujing Qian, Hang Song, Yurong Tuo, Feiyue Xiao, Li Zhang, Chan Zhao, Li Cai, Ling Chen, Wei Chi, Rongping Dai, Liqun Du, Liping Du, Shifeng Fang, Lei Feng, Ling Gao, Xiaofeng Xie, Lin Liu, Xiaoli Liu, Peirong Lu, Haifeng Mei, Zhenhai Nie, Hong Ning, Yanyun Shi, Yong Tao, Hong Wang, Yuqin Wang, Huanwen Wu, Xinyi Wu, Yanfang Wu, Dan Xie, Huizhuo Xu, Liu Yang, Caiyun You, Lianming Zhang, Xiaomin Zhang, Hui Zhong, Huifang Zhou, Wenjuan Zhuang, Hongfeng He, Dongrui Peng, Huayu Zhang, Yuelun Zhang, Qing Chang, Wenbin Wei, Daobin Zhou

**Affiliations:** 1 Department of Ophthalmology, Peking Union Medical College Hospital, Chinese Academy of Medical Sciences and Peking Union Medical College, Beijing, 100730 China; 2 Beijing Key Laboratory of Fundus Diseases Intelligent Diagnosis & Drug/Device Development and Translation, Beijing, 100730 China; 3 Key Laboratory of Ocular Fundus Diseases, Chinese Academy of Medical Sciences, Beijing, 100730 China; 4 Ophthalmology Department, Beijing Tongren Hospital, Capital Medical University, Beijing, 100005 China; 5 Zhongshan Ophthalmic Center, Sun Yat-Sen University, Guangzhou, 510623 China; 6 Research Unit of Evidence-Based Evaluation and Guidelines, Chinese Academy of Medical Sciences (2021RU017), School of Basic Medical Sciences, Lanzhou University, Lanzhou, 730000 China; 7 WHO Collaborating Center for Guideline Implementation and Knowledge Translation, Lanzhou, 730000 China; 8 Lanzhou University GRADE Center, Lanzhou, 730000 China; 9 Department of Hematology, Peking Union Medical College Hospital, Chinese Academy of Medical Sciences and Peking Union Medical College, Beijing, 100730 China; 10 Ophthalmology Medical Center, The First Affiliated Hospital of Chongqing Medical University, Chongqing Key Laboratory for the Prevention and Treatment of Major Blinding Eye Diseases, Chongqing Branch (Municipality Division) of National Clinical Research Center for Ocular Diseases, Chongqing, 400016 China; 11 Department of Ophthalmology, Joint Research Laboratory for Ocular Immunology and Retinal Injury Repair, The First Affliated Hospital of Zhengzhou University, Henan International, Henan Province Eye Hospital, Zhengzhou, 450052 P.R. China

**Keywords:** Vitreoretinal lymphoma, Central nervous system lymphoma, Diagnosis, Treatment

## Abstract

Vitreoretinal lymphoma (VRL) is often a diffuse large B-cell lymphoma in nature, and patients may have or eventually develop central nervous system lymphoma, which frequently leads to a poor prognosis. Currently, there are no international or domestic clinical guidelines specifically for the diagnosis and treatment of VRL, and no standardized diagnostic procedures or treatment evaluation systems for this disease. VRL is clinically characterized by prominent vitreous opacities, multiple lesions beneath the retinal pigment epithelium or subretinal, and intraretinal infiltration, making it one of the most common masquerade syndromes in ophthalmology. To promote early diagnosis and standardized treatment of VRL, the Ocular Immunology Group of the Chinese Medical Association Ophthalmology Branch has developed "Clinical Guidelines for the Diagnosis and Treatment of Vitreoretinal Lymphoma in Chinese Patients (2024)", based on extensive references to diagnosis, treatment experiences and relevant clinical recommendations. The working group systematically reviewed and comprehensively summarized the latest research evidence from both domestic and international sources. Using the Oxford Evidence Level System, we assessed the quality of evidence and the strength of recommendations. This guideline provides crucial academic references and clinical practice guidance for the diagnosis and treatment of VRL patients. This guideline, including VRL diagnostic methods, processes, and treatment recommendations is suitable for clinical practice in China and is intended to assist ophthalmologists in clinical diagnosis and treatment of VRL.

## Background

Vitreoretinal lymphoma (VRL) is a type of intraocular lymphoma and the most common masquerade syndrome of the eye [1]. In primary vitreoretinal lymphoma (PVRL), lymphoma cells initially exist only in the eye involving the retina, vitreous, or optic nerve without evidence of intracranial or cerebrospinal fluid (CSF) involvement [2]. Up to 15%–25% of patients with primary central nervous system lymphoma (PCNSL) have or eventually develop VRL [3, 4] while 42%–92% of PVRL patients develop central nervous system (CNS) diseases within an average of 8 to 29 months, and the prognosis is poor in most cases [5]. The etiology of PVRL is unclear, and there is no accurate epidemiologic information. However, the incidence of PVRL in the elderly has continually increased over the last two decades [6], possibly related to advances in disease diagnostic methods that have improved diagnostic sensitivity.

Over 95% of patients with PVRL have diffuse large B-cell lymphoma (DLBCL), which often masquerades as posterior or intermediate uveitis [7, 8]. Most patients have bilateral disease, but the two eyes may have different sequences and symptoms, mostly presenting with blurred vision, decreased visual acuity, floaters, etc. Obvious vitreous haze and multiple subretinal pigment epithelium (RPE), subretinal, or intraretinal infiltrative lesions are its characteristic manifestations. Most of the patients are diagnosed with various types of uveitis at disease onset and are treated with glucocorticoids, which may be effective initially, but this is a protracted disease. Many patients are not definitively diagnosed until they present with CNS symptoms. In fact, it takes an average of 6 to 40 months from the onset of ocular symptoms to definitively diagnosing VRL [9].

There are currently no international clinical guidelines for the diagnosis and treatment of VRL, and only a few PCNSL guidelines briefly address VRL, as well as sporadic diagnostic and therapeutic recommendations [8, 10–13]. Since there is no standardized diagnostic procedure and efficacy evaluation system for VRL, clinical diagnosis and treatment are particularly challenging for this disease. Based on domestic and international diagnostic and treatment experiences and relevant treatment recommendations, the Ocular Immunology Group of the Chinese Medical Association Ophthalmology Branch has taken the lead in formulating the “Clinical Guidelines for the Diagnosis and Treatment of Vitreoretinal Lymphoma in Chinese Patients (2024)”, which is intended to be used as a reference for ophthalmologists in the clinical diagnosis and treatment of this disease. This guideline is applicable to DLBCL. Rare forms of non-DLBCL lymphoma are outside the scope of this guideline.

### Methodologies

The guidelines were developed following the WHO Handbook for Guideline Development (2nd Edition) and the Guiding Principles for the Development/Revision of Clinical Diagnosis and Treatment Guidelines in China (2022 Edition) [14, 15]. Additionally, the guidelines adhere to the Reporting Items for Practice Guidelines in HealThcare (RIGHT) statement for reporting [16].

#### Initiating organization

The development of this guideline was initiated by the Ocular Immunology Group of the Chinese Medical Association Ophthalmology Branch. Methodological support was provided by The Guidelines and Standards Research Center of the Institute of Health Data Science at Lanzhou University.

#### Guideline registration

This document has been registered on the Practice guideline REgistration for transPAREncy (PREPARE) platform (registration number: PREPARE-2023CN370) [17].

#### Guideline users and target population

It is developed for ophthalmology clinicians and is specifically aimed at patients diagnosed with or suspected of VRL.

#### Guideline development working group

A multidisciplinary working group comprising experts in ophthalmology, pathology, hematology, nursing and guideline methodology was set up. The working group is structured into several subcommittees, including a steering committee, a secretarial group, a consensus expert group, a methodology group, and an external review group [18]. All members of the guidelines working group are required to finish a questionnaire to disclose any relevant financial or academic conflicts related to the formulation of these guidelines that they may have had over the past three years [19]. Upon review by the Steering Committee, one expert has a financial conflict and is excluded from the Delphi investigation stage. The remaining members of the working group declare no conflicts of interest.

#### Determination of clinical questions

The Secretarial Group initiated the process by compiling a list of clinical questions based on preliminary discussions with experts. This organized list of clinical questions was then distributed to the members of the Consensus Expert Group via a questionnaire. The questionnaire required the experts to assign a score for each clinical question based on the clinical importance. The score ranged from 1 to 5, where 5 indicated that the question was critical for decision-making and recommendation, while 1 suggested that it was not of immediate concern. The experts could also propose clinical questions which were considered as important for the diagnosis and treatment through the questionnaire. Utilizing the questionnaire results, the Secretarial Group prioritized the clinical questions by their level of importance and presented a list of supplementary clinical questions. The Steering Committee then made the final determination on which clinical questions should be incorporated into the guidelines.

#### Evidence search

The Methodology Group searched for Chinese and English databases including Medline (via PubMed), Cochrane Library, Web of Science, Embase, the China Knowledge Network Database (CNKI), Wanfang Data and the China Biomedical Literature Service System (CBM). The search strategy was developed using terms “vitreoretinal lymphoma”, “intraocular lymphoma”, “diffuse large B-cell lymphoma” and other related synonyms. We included systematic reviews, meta-analyses, randomized controlled trials and observational studies related to VRL, which were published in Chinese or English journals before November 2023. Additionally, a hand search was performed to track most recent publications.

#### Evidence grading

The Methodology Group adopted the Oxford Center for Evidence-Based Medicine (OCEBM) Levels of Evidence Criteria (2009 Edition) to conduct evidence assessment and grading recommendations [20]. Given the limited clinical evidence currently available for certain diagnostic and treatment recommendations of VRL, this guideline develops four recommendations for questions which are now lacking evidence but need guidance in clinical practice by expert consensus. The detailed Oxford grading criteria are provided in Table 1.

**Table 1:** Oxford Center for Evidence-Based Medicine (OCEBM) Levels of Evidence Criteria (2009 Edition)

Recommendations	Level of evidence	Description
A	1a	SR based on RCTs (with homogeneity)
	1b	Individual RCT studies
	1c	"All or none" evidence
B	2a	SR based on cohort studies (with homogeneity)
	2b	Single cohort studies (including low-quality RCTs e.g., less than 80% follow-up)
	3a	SR based on case-control studies (with homogeneity)
	3b	Single case-control study
C	4	Case reports, low-quality cohort or case-control studies
D	5	Expert opinion or comment, bench research or “first principles”

SR = systematic review; RCT = randomized controlled trial

#### Recommendations formation

The Secretarial Group summarized the available evidence and drafted preliminary recommendations, considering the certainty of evidence, cost-effectiveness, patient value and preferences, and other factors. For each recommendation, the Secretarial Group conducted one round of Delphi survey. The Consensus Expert Group participated in this process by completing the Delphi survey, selecting “agree”, “disagree” or “uncertain” for each recommendation. The consensus level, defined as the agreement rate, for all recommendations exceeded 80% in this survey. With such a high level of consensus achieved, the Secretarial Group organized an online consensus meeting in April 2024. During this meeting, the Secretarial Group addressed the comments received, made revisions according to experts’ opinions and formulated the final recommendations.

#### Guideline writing, external review, and publication

The Secretariat Group summarized the final recommendations, drafted the guidelines, and submitted them to the External Review Group. The Secretariat Group revised the draft guidelines based on the comments from the external reviewers and submitted the document to the Steering Committee for approval.

#### Dissemination and updating

After the guidelines are published, the working group plans to disseminate it by propagandizing online and offline academic conferences at the national level to promote its adoption and use. Concurrently, the working group would maintain a regular literature search and evaluation. The working group will update the guidelines at appropriate periods, following strict updating methodologies and processes [21].

### Clinical questions and recommendations

#### Clinical question 1: what are the ocular signs and symptoms in patients with VRL?

Recommendation 1: The first symptom of VRL includes floating objects of the eyes or painless loss of vision. (4, C; Consensus level 96.55%).

Recommendation 2: The most common sign of VRL is vitreous cellular opacity, and it may be the only positive sign for some patients. (2b, B; Consensus level 96.55%).

Recommendation 3: Other signs include intraretinal, subretinal, or sub-RPE infiltrative lesions, optic disc infiltration, retinal hemorrhage, exudative retinal detachment, retinal vasculitis, and anterior chamber reaction. (4, C; Consensus level 93.10%).

#### Basis of recommendation and evidence summary

The initial symptoms of VRL vary considerably. Most cases involve both eyes, but the onset can be sequential with varying severity. Patients usually experience blurred vision or floaters and may also present with painless vision loss [22–27]. The degree of visual impairment varies. Most patients can maintain relatively good visual function though some may experience visual impairment and even vision loss [26].

The most common manifestation of VRL is vitreous haze, which can appear as a curtain-like or a snowball-like opacity. Some patients may only exhibit vitreous opacity as a positive sign [22–27]. Retinal involvement is evidenced by the intraretinal, subretinal, or sub-RPE infiltrative lesions. There may also be signs of retinal vasculitis. In more severe cases, there could be optic disc infiltration, disc hemorrhage or edema, and even exudative retinal detachment [22–27]. Other manifestations include anterior segment reaction, as evidenced by keratic precipitates or anterior chamber cells. Rare signs include secondary glaucoma, vitreous hemorrhage, and hypopyon [22, 23, 26, 27].

#### Clinical question 2: what ophthalmic imaging examinations should be performed for patients with VRL?

Recommendation 4: For VRL patients, the following ophthalmic imaging examinations are recommended: fundus photography [preferably ultra-wide field (UWF) fundus imaging], optical coherence tomography (OCT), and ocular B-scan ultrasonography. Depending on the condition, additional examinations such as fundus autofluorescence, fluorescein fundus angiography (FFA), indocyanine green angiography (ICGA), and OCT angiography (OCTA) may be needed. Orbital magnetic resonance imaging (MRI) is not recommended. (1b, A; Consensus level 96.30%).

#### Basis of recommendation and evidence summary

For suspected VRL patients, it is recommended to complete ophthalmic imaging tests as they are crucial diagnostic tools for this disease. Moreover, these are routine, cost-effective, and practicable methods in clinical practice.

Fundus photography (preferably using UWF) shows vitreous opacities in 92% to 100% of the affected eyes, characterized by clustered or patchy gray-white cells in the vitreous [28, 29]. A retrospective study published in 2019 categorized vitreous opacities in UWF images of 26 eyes with VRL into three types: aurora borealis pattern, string of pearls pattern, and nonspecific pattern [29]. Retinal lesions may include retinal infiltration and sub-RPE deposits, appearing as multiple subretinal yellow-white lesions, flat or elevated, sometimes merging, with some lesions showing characteristic ‘leopard skin’ pigmentation [22, 28, 30, 31]. Other abnormalities include retinal hemorrhages, optic nerve infiltration, necrotizing retinitis-like changes, and exudative retinal detachments [22, 30, 31].

OCT findings are mainly categorized into three types of abnormalities: (1) Vitreous abnormalities, such as vitreous cells [22, 32–34] and cell aggregation in posterior vitreous cortex [28]; (2) Retinal abnormalities, including retinal infiltration [22, 28, 32, 33, 35, 36], particularly vertical hyperreflective lesions involving the entire neuroretina [37, 38], subretinal nodular or band-like homogeneous hyperreflective lesions [22, 28, 32, 33, 35, 36], irregular ellipsoid zone [36, 37], unclear outer retinal structure [32, 37, 39], retinal thickening [37, 40], subretinal fluid [22, 28, 32, 37, 39], and preretinal deposits [33, 37]; (3) RPE abnormalities, including RPE infiltration [22, 33], RPE detachment [22, 28, 32, 39], sub-RPE nodular hyperreflective lesions [39, 41, 42], and sub-RPE homogeneous hyperreflective lesions [33, 35, 37, 40]. Macular edema can occur but is rare. Macular edema and epiretinal membranes usually occur in long-term cases. OCTA has limited applications in VRL. In 2022, a retrospective study of 23 cases with 35 eyes found that 34.3% of the affected eyes showed punctate points or confluent bands surrounding retinal vessels in en-face OCTA, corresponding to hyperreflective lesions throughout the retina in OCT, which may reduce or disappear after treatment [43].

Ultrasound imaging in VRL patients shows vitreous opacities, posterior vitreous detachment, retinal detachment, and irregular hypoechoic lesions under the retina. Doppler imaging may show detectable blood flow signals within some lesions [44]. Compared to uveitis, VRL patients have a lower incidence of vitreoretinal adhesion on B-scan ultrasound. Thickening or occupying lesions of the retina and centrifugal condensation pattern of vitreous haze might be suggestive of VRL [45].

The proportion of abnormal fundus autofluorescence varies greatly in different studies, ranging from 61% to 100%, which may be related to whether the lesion involves the retina. Leopard-like changes, i.e., mixed spots of high and low autofluorescence are the most common pattern [22, 28, 41]. Hyper-autofluorescent lesions can be focal or reticular [28], highly correlated with RPE thickening and large RPE detachment on OCT [35], indicating lesion activity [41], which improve with treatment [31, 39].

From 2002 to 2022, the FFA and ICGA findings of VRL were summarized in nine retrospective studies. Only two studies were published in the last five years [22, 35]. The most common FFA findings are RPE abnormalities, including non-leaking hyperfluorescent spots, “leopard-like” hypofluorescence or small hypofluorescent spots, and late staining, related to lymphoma cells between the RPE and Bruch’s membrane [28, 30, 35, 41, 42, 46, 47]. There may be vascular leakage, staining [22, 28, 35, 42], with less frequent optic disc edema and cystoid macular edema [22, 28, 30, 35]. ICGA shows hypofluorescent lesions, predominantly in the posterior pole, but these lesions are noticeably fewer compared to FFA [28, 47].

Orbital MRI has low sensitivity for VRL, making it less useful for differentiating VRL from uveitis or ocular melanoma.

In summary, for patients suspected of VRL, it is recommended to complete fundus photography (preferably UWF), OCT, and ocular B-scan ultrasound. It is important to note that ophthalmic imaging characteristics cannot predict the occurrence and progression of CNS lesions [48].

#### Clinical question 3: what systemic examinations should be conducted for patients with VRL?

Recommendation 5: Patients with suspected or confirmed VRL are recommended to undergo CNS imaging, including enhanced MRI of the brain and brainstem. (4, C; Consensus level 100%).

Recommendation 6: Patients with suspected or confirmed VRL are recommended to undergo a lumbar puncture to check the CSF for interleukin (IL)-10/IL-6 levels. (1b, A; Consensus level 93.33%).

Recommendation 7: Confirmed VRL patients are recommended to have a whole-body positron emission tomography (PET-CT) imaging workup. (Expert Opinion; Consensus level 87.50%).

#### Basis of recommendation and evidence summary

VRL is generally classified as a non-Hodgkin's lymphoma (NHL) with embryological connections to CNS lymphomas [49] and has a poor prognosis if CNS involvement is present [50]. Therefore, comprehensive examinations are crucial for disease evaluation and treatment assessment.

In VRL patients, most CNS lesions are found in the forebrain while the cerebellum and brainstem are more commonly affected in the hindbrain lesions [4]. Enhanced MRI is currently the preferred imaging modality for diagnosis and assessment [51, 52]. Typically, enhanced MRI is performed before aqueous humor or vitreous biopsy. If intracranial lesions are found, a neurosurgeon consultation is needed to determine whether to perform intracranial lesion biopsy.

Research indicates that CSF IL-10 levels can aid in diagnosing PCNSL and VRL, with sensitivity ranging from 63.9% to 95.7% and specificity from 94.1% to 100% [53–56]. Elevated IL-10 levels in CSF are observed in 89.7% of VRL patients. Patients with intracranial lesions showed higher CSF IL-10 levels compared to those without, suggesting that IL-10 serves as a biomarker for CNS involvement [57].

The *MYD88* L265P mutation is a unique nonsynonymous point mutation found in B-cell malignancies [58]. Detection of *MYD88* L265P in CSF can be a molecular marker for diagnosing VRL, with some patients having co-mutations with *CD79B* [56, 59]. The sensitivity and specificity of CSF *MYD88* L265P for diagnosing VRL are 63.2% and 100%, respectively, highlighting its potential for diagnosis and monitoring of VRL [56, 60].

CSF cytology and flow cytometry can be useful for suspected CNS involvement in VRL, but shows low sensitivity. At least 3–10 mL of CSF is required, and only a few cells exist, which is not enough for flow cytometry analysis, and may require multiple lumbar punctures, limiting its clinical utility [51, 61, 62].

PET-CT is sensitive to PCNSL lesions and can delineate tumor extent and surrounding tissue involvement [63]. A meta-analysis shows PET-CT has a sensitivity of 87% and specificity of 85% for diagnosing PCNSL [64]. The International Primary CNS Lymphoma Collaborative Group recommends MRI and PET imaging for PCNSL, including VRL, to improve diagnostic accuracy and treatment response evaluation [65]. Whole-body PET-CT is recommended for confirmed VRL patients, especially at initial diagnosis or recurrence, to exclude systemic involvement and enhance diagnostic accuracy, though its cost-effectiveness needs further research [63].

Some studies have identified hematological features in VRL patients, such as abnormalities in serum sIL-2R, microRNA, and IgA levels [66–68]. However, these findings are relatively isolated and need further validation. Related hematological tests are not currently recommended.

#### Clinical question 4: what intraocular fluid tests should VRL patients undergo?

Recommendation 8: For suspect VRL patients, an initial aqueous humor analysis for IL-10 and IL-6 concentration to screen for the disease can be performed. (2b, B; Consensus level 96.77%).

Recommendation 9: For patients with a high suspicion of VRL, diagnostic vitrectomy is recommended. During the procedure, vitreous samples should be collected for cytopathology, IL-10 and IL-6 concentration (if aqueous humor IL-10 and IL-6 testing was not performed), gene rearrangement, and flow cytometry. Testing for the *MYD88* L265P mutation should be considered if feasible. (2b, B; Consensus level 93.55%).

Recommendation 10: For patients with a high suspicion of VRL and inconclusive results from the initial diagnostic vitrectomy, a repeat diagnostic vitrectomy is recommended, preferably in the contralateral affected eye. (4, C; Consensus level 100%).

#### Basis of recommendation and evidence summary

IL-10 is a critical cytokine biomarker for VRL originating from B cells. The concentration of IL-10 in the aqueous humor and/or vitreous samples, or the IL-10/IL-6 ratio, is commonly used for screening or diagnosing VRL [69–74]. IL-10 levels are typically significantly elevated in VRL aqueous humor and vitreous samples compared to non-VRL intraocular fluid samples [69–74]. However, there is considerable variability in the absolute values of IL-10 concentrations across different studies. A multicenter study across 23 hospitals in France, Belgium, and Switzerland introduced the Interleukin Score for intra-Ocular Lymphoma Diagnosis (ISOLD), whereby intraocular fluid IL-10 and IL-6 concentrations were used for VRL diagnosis [72]. Despite its high diagnostic efficacy, its complexity and limited improvement over the IL-10/IL-6 ratio have led to its current non-recommendation [69]. Most studies show no significant difference between aqueous humor and vitreous samples for diagnosis [69, 72, 73], but aqueous humor samples are easier to obtain. Thus, the IL-10/IL-6 ratio in aqueous humor can be used for screening and follow-up of suspected VRL patients [71, 73]. Methods for detecting IL-10 and IL-6 concentrations in aqueous humor include enzyme-linked immunosorbent assay (ELISA), cytometric bead array, and Luminex xMAP. Given the limited volume of aqueous humor available clinically, the latter two methods may be more suitable. After intravitreal methotrexate (MTX) treatment, IL-10 levels in the intraocular fluid may decrease or normalize with disease remission, and a subsequent rise in IL-10 levels may indicate disease recurrence. Therefore, IL-10 in intraocular fluid can also serve as a prognostic indicator and a measure of treatment efficacy [75, 76].

Diagnostic vitrectomy is recommended for patients who are highly suspected of VRL if common causes of chronic uveitis are ruled out [77, 78]. Although vitreous cell pathology is the recognized gold standard for diagnosing VRL, its high false-negative rate necessitates the use of additional diagnostic methods, including IL-10/IL-6 ratio or IL-10 concentration [74, 79], immunoglobulin heavy chain (IgH), light chain, and T-cell receptor (TCR) gene rearrangement detection [74, 77, 80], flow cytometry [77, 79–81], and chorioretinal biopsy [78]. A retrospective study by the NEI in the US (1998–2010) involving 214 patients with clinically suspected PVRL found that immunoglobulin heavy and light chain and TCR gene rearrangements had the highest diagnostic value. Cytological analysis showed higher specificity and positive predictive value for PVRL patients, while cytokine analysis had higher sensitivity and negative predictive value [82].

Recent studies have demonstrated the clinical value of testing for *MYD88* L265P mutations in aqueous humor or vitreous specimens. A UK study in 2015 (n = 69) showed that *MYD88* mutation detection could increase the sensitivity of traditional vitreous testing for VRL from 62% to 90.5% and the negative predictive value from 85% to 96% [83]. An Italian study in 2019 found a 100% positivity rate for *MYD88* L265P mutations in vitreous from eight VRL patients and a 75% positivity rate in aqueous humor specimens [84]. A Chinese study in 2020 using droplet digital polymerase chain reaction (PCR) found an 80% positivity rate for *MYD88* L265P mutations in vitreous specimens [85]. An Indian study in 2020 using PCR amplification and restriction fragment length polymorphism methods found sensitivities and specificities of 88.9% and 91.6%, respectively, in VRL patients [86]. A study in 2022 using next-generation sequencing identified a 74% mutation rate of *MYD88* in VRL, predominantly L265P mutations [87]. Given the high *MYD88* mutation rate and its prognostic significance in PCNSL, *MYD88* L265P mutation testing should be considered when feasible.

Preliminary studies on aqueous or vitreous cell-free DNA (cfDNA) sequencing show clinical value. Gu et al. performed cfDNA sequencing on the vitreous from patients with VRL or intraocular inflammatory disease and found that *MYD88* and/or *ETV6* mutations with sensitivities and specificities were 91.3% and 95.0%, respectively. *CD79B* mutations were associated with higher intraocular IL-10 levels, *CARD11* mutations with earlier onset, and patients with intracranial involvement had more multisite mutations in the *BTG2* gene [88]. Chen et al. used cfDNA gene mutation analysis for intraocular fluids and found a 100% sensitivity for diagnosing PVRL, with the most common gene mutations being* PIM1*, *MYD88*, *ETV6*, *IRF4*, and *CD79B* [89]. Aqueous humor can also be used for analyzing tumor genomic information, demonstrating clinical utility in diagnosing and monitoring VRL treatment [74, 90].

#### Clinical question 5: how should diagnostic vitrectomy be performed for patients with VRL?

Recommendation 11: A three-port vitrectomy with a cutting rate of 600 to 1200 cuts per minute is recommended. (4, C; Consensus level 96.30%).

Recommendation 12: Aspirate 1.5 mL to 2.0 mL of vitreous fluid and send it for testing within 1 h. During specimen collection, air infusion can be used to maintain intraocular pressure as needed, and after opening the intraocular infusion, diluted vitreous fluid is to be collected for various tests. (3b, B; Consensus level 96.30%).

Recommendation 13: Vitreous samples for cytological examination should be quickly transferred to cell culture medium or cell preservation solution to maintain cell viability. (4, C; Consensus level 88.89%).

Recommendation 14: Patients with negative cytological results, but progressive retinal changes should undergo subretinal puncture or chorioretinal biopsy to aid in diagnosis. (4, C; Consensus level 85.19%).

#### Basis of recommendation and evidence summary

Three-port vitrectomy is the most commonly used surgical method for diagnosing VRL. Available gauges include 20G, 23G, 25G, and 27G. Compared to the 20G vitrectomy, which involves conjunctival incisions, scleral puncture, and scleral sutures, the 25G transconjunctival sutureless vitrectomy (25G-TSV) offers advantages such as reduced surgery time and postoperative pain, faster wound healing, and quicker vision recovery. Although 25G-TSV may lead to rare events like bubble formation at the scleral incision and transient early postoperative hypotony, there is no difference concerning severe complications such as endophthalmitis between 25G-TSV and traditional 20G vitrectomy. Other postoperative complication risks, such as retinal detachment and vitreous hemorrhage, are also lower with 25G-TSV [80].

Diagnostic vitrectomy is a safe method for diagnosing VRL. A 2020 meta-analysis reviewed 16 studies and found a 46% improvement in vision among VRL patients after vitrectomy, highlighting the need to minimize additional damage during the procedure [91]. The frequencies of postoperative complications including cataract and retinal detachment were 19% and 5%, respectively [91]. From 2007 to 2023, multiple studies described aspirating 1.5 mL to 2.0 mL of vitreous fluid under air infusion to maintain intraocular pressure for laboratory examination within an hour [57, 73, 92–94]. In multiple studies from 2004 to 2019, it was mentioned that vitreous samples were sent for cytological examination in cell culture medium, with the most commonly used being RPMI-1640 containing 10% fetal bovine serum [73, 92, 95, 96]. A 2019 study indicated that subretinal puncture or chorioretinal biopsy could assist in diagnosing when vitreous samples are negative but progressive retinal changes are present [97]. A 2020 meta-analysis included five studies with both retinal and choroidal biopsies, and confirmed clinical diagnoses of lymphoma [91].

#### Clinical question 6: is there currently a gold standard for diagnosing VRL?

Recommendation 15: The gold standard for diagnosing VRL is pathological examination of the vitreous, retinal, or optic nerve. The diagnosis of VRL is confirmed definitively by the presence of malignant lymphoma cells in ocular samples. (1a, A; Consensus level 96.77%).

#### Basis of recommendation and evidence summary

According to existing literature and international consensus, evaluating the presence of malignant lymphoma cells through cytopathology and immunohistochemical staining is the most reliable indicator for diagnosing VRL [8]. Detection of these malignant cells in ocular fluids (vitreous or aqueous humor) or subretinal tissues is widely accepted as the gold standard for VRL diagnosis. Typically, tumor cells on smears are large, atypical cells, approximately 2 to 5 times the size of small lymphocytes, characterized by large, irregular nuclei, prominent nucleoli, and basophilic cytoplasm [98]. Immunohistochemical staining for CD20 confirms B-cell lymphoma, while Ki67 indicates proliferation activity.

Diagnosing VRL through pathology can be challenging due to the limited volume of ocular samples, the low cell density in vitreous fluid, and the extreme fragility of lymphoma cells. This results in varying positive rates across different medical institutions. A 2022 meta-analysis of 87 studies involving 1484 patients found that cytological examination of lymphoma cells remains the gold standard with the highest specificity, though sensitivity is around 80% [99]. A 2012 Japanese study reported a 44.5% positivity rate in 164 patients with intraocular lymphoma [27]. In 2023, Zhang et al. conducted diagnostic vitrectomy on 43 eyes of 40 VRL patients and found malignant or atypical cells in 81.4% (35/43) of the cases [94]. In 1997, Davis et al. increased the diagnostic rate of PVRL from 30% to 70% through immunohistochemistry [100]. Most PVRL cases originate from B cells, and immunohistochemistry shows positive results for B cell markers such as CD19, CD20, and CD22, with CD20 being the most commonly used marker. The biomarkers of T-cell lymphoma include CD3 [101]. Another method to improve detection rates is the cell block technique, where diluted vitreous cell particles are fixed with 10% formalin, embedded in paraffin, and examined immunohistochemically. Kase et al. found that the positive rate of atypical cells detected using the cell block method was 93.3% compared with 35.7% with routine smears [102]. Although molecular testing has advanced to address the low sensitivity of cytology, pathological evidence remains the gold standard for confirming VRL diagnosis.

#### Clinical question 7: how do we diagnose suspected VRL patients with negative vitreous cytology results?

Recommendation 16: For suspected VRL patients with negative vitreous cytology results, additional tests should be considered: intraocular fluid IL-10/IL-6 ratio, gene rearrangement, *MYD88* L265P gene mutation, and flow cytometry. (1a, A; Consensus level 96.77%).

Recommendation 17: When vitreous cytology results are negative, but the patient has typical ocular findings such as vitreous opacities and/or subretinal infiltrative lesions, VRL can be diagnosed in cases with two or more of the following tests: intraocular fluid IL-10/IL-6 > 1, a positive result of flow cytometry and gene rearrangement. (4, C; Consensus level 96.77%).

Recommendation 18: If vitreous cytology results are negative but the patient has typical ocular findings, VRL can be diagnosed when intraocular fluid IL-10/IL-6 > 1 and *MYD88* L265P gene mutation is positive. (Expert Opinion; Consensus level 93.55%).

Recommendation 19: When the patient has typical ocular findings and only intraocular fluid IL-10/IL-6 > 1 is present, if lymphoma is detected in the CNS or other organs, VRL should be diagnosed. If systemic involvement is not detected, suspected VRL should be considered and the patient monitored closely. (Expert Opinion; Consensus level 96.77%).

#### Basis of recommendation and evidence summary

IL-10, a cytokine expressed by malignant B lymphocytes, is significantly elevated in the vitreous and aqueous humor of VRL patients [57, 73, 99]. Increased IL-6 levels in intraocular fluid can result from inflammation, and the IL-10/IL-6 ratio is used to differentiate VRL from uveitis. An IL-10/IL-6 ratio > 1 indicates VRL (sensitivity 81%–92%, specificity 100%), with a higher ratio providing greater diagnostic significance [56, 99, 103]. Non-B-cell lymphomas, such as mantle cell or NK/T-cell lymphomas, do not show elevated IL-10 levels [104]. In cases of widespread and severe RPE or retinal infiltration in PVRL, IL-6 levels may arise due to the disruption of the blood-retinal barrier or secondary inflammation, leading to a reduced IL-10/IL-6 ratio. Thus, the absolute IL-10 values (aqueous humor > 50 pg/mL, vitreous > 400 pg/mL) are crucial for diagnosis [73, 103, 104].

Due to the fragility of lymphoma cells, cell fragmentation during collection and examination may result in false-negative cytology results. Collecting cellular debris from centrifuged intraocular fluid and performing DNA-related tests can offer higher sensitivity. As an intracellular signaling protein often mutated in DLBCL, the diagnostic utility of *MYD88* L265P mutation detection in the vitreous or aqueous humor of patients with VRL had a sensitivity of 67% to 89%, and specificity of 100% [84, 105–107]. Studies show that *MYD88* L265P gene mutation is not detected in eyes with complete remission but is present in recurrent cases, indicating the presence of intraocular tumor cells [106].

Positive IgH or light chain gene rearrangements in B lymphocytes and TCR gene rearrangements in T lymphocytes are molecular characteristics of VRL. The positivity rate of IgH gene rearrangement in B lymphocytes among VRL patients is 71%–84% [99, 103, 104], while TCR gene rearrangement in T lymphocytes is nearly 100% (T-cell lymphoma is rarer with fewer published studies, each with fewer than five cases) [99, 104].

Flow cytometry is another technique for immunophenotyping intraocular cells. B cell-specific surface markers (such as CD19 and CD20) support the diagnosis and classification of B-cell lymphoma [7], with 63%–82% sensitivity and 97%–100% specificity [81, 98]. Flow cytometry requires a large number and intact vitreous lymphocytes for analysis and insufficient cell quantity can lead to analysis failure or false negatives, limiting its clinical application [106].

In 2020, a case–control study from Japan used PCR to detect immunoglobulin gene rearrangement and cytokine concentrations in vitreous samples from 22 patients with clinically suspected VRL and 10 controls with acute retinal necrosis or cytomegalovirus retinitis. It was found that a diagnosis of VRL could be made in patients with the typical signs of VRL if gene rearrangement or IL-10/IL-6 > 1 was positive, with both sensitivity and specificity of 100% [108]. In 2023, Zhang et al. questioned this diagnostic standard [109] and proposed that when patients have typical clinical signs of VRL and negative vitreous cytology results, diffuse large B-cell PVRL can be diagnosed in cases with two or more of the following tests: intraocular fluid cytokine IL-10/IL-6 > 1, a positive result of vitreous IgH and/or Igκ, Igλ gene rearrangement, and a positive result of vitreous flow cytometry showing B-cell clonality. The sensitivity and specificity were 97.5% and 100%, respectively [94].

In summary, for suspected VRL patients with negative vitreous cytology results, VRL can be diagnosed if intraocular fluid IL-10/IL-6 > 1 and at least one of the following tests is positive: flow cytometry, gene rearrangement, or *MYD88* L265P gene mutation. If intraocular fluid IL-10/IL-6 > 1 is not present, but flow cytometry and gene rearrangement are positive, VRL can also be diagnosed. Preliminary exploration of aqueous humor or vitreous cfDNA sequencing suggests potential clinical value, but further studies are needed to establish it as a diagnostic standard for VRL.

#### Clinical question 8: how can we diagnose suspected VRL patients with a history of CNS lymphoma?

#### Clinical question 9: how can we diagnose patients with clinical manifestations of VRL after being diagnosed with DLBCL in other organs?

Recommendation 20: If ocular symptoms or signs related to VRL are present for patients with a history of DLBCL in the CNS or other organs, levels of IL-10 and IL-6 in the aqueous humor should be determined. If the IL-10/IL-6 ratio is greater than 1, VRL can be diagnosed. (2b, B; Consensus level 96.67%).

#### Basis of recommendation and evidence summary

The eye is the most commonly affected organ in PCNSL [110]. In clinical practice, over 15% of PCNSL patients develop ocular involvement [4, 111, 112]. Therefore, for patients with a diagnosis of DLBCL in the CNS or other organs, if symptoms such as vision loss, dust-like or curtain-like vitreous opacities, or retinal yellow-white infiltrative lesions occur, especially if the anti-inflammatory treatment is ineffective, VRL should be strongly suspected [111].

Histopathological examination is the gold standard for diagnosis, whereas elevated IL-10 or an IL-10/IL-6 ratio > 1 serves as a screening indicator for intraocular DLBCL. Chan et al. suggested that for patients with a confirmed diagnosis of PCNSL or DLBCL in other organs, intraocular tissue biopsy may not be considered [4, 113].

However, this recommendation does not negate the diagnostic value of diagnostic vitrectomy in confirming VRL. After discussing the condition with the patient, if they request a vitreous biopsy or require therapeutic vitrectomy, vitreous cell pathology should be considered.

#### Clinical question 10: what are the recommendations for ocular local treatment in patients with confirmed VRL?

Recommendation 21: For patients with bilateral involvement or concurrent CNS involvement, it is recommended to combine ocular treatment with systemic therapy. For those with unilateral involvement, ocular treatment alone may be considered, provided there is close monitoring for potential CNS involvement. (1b, A; Consensus level 96.67%).

Recommendation 22: The preferred local treatment is intravitreal MTX injection. Alternative treatments include intravitreal rituximab injection and local radiation therapy. (1b, A; Consensus level 96.67%).

Recommendation 23: Effectiveness of local treatment should be assessed based on clinical presentation, imaging results, and intraocular fluid cytokine testing. (1b, A; Consensus level 100%).

#### Basis of recommendation and evidence summary

Both ocular and systemic therapies are used for VRL. Ocular treatment options include intravitreal MTX injection, intravitreal rituximab injection, and local radiation therapy. A unified treatment protocol has not yet been achieved. A 2018 Mayo Clinic review recommended local treatment alone for unilateral cases and combination of local and systemic therapy for bilateral cases or those with ocular and brain involvement [114]. Studies show no significant difference in recurrence rates between local with and without systemic treatments for unilateral cases, though research is limited and retrospective [22, 114]. The 2007 International PCNSL Collaborative Group study indicated that ocular treatment alone does not increase the risk of CNS relapse [115]. The “Chinese expert consensus on the management of PCNSL” suggests a combination of systemic and local treatment for PVRL patients or PCNSL with ocular involvement. For unilateral PVRL, local treatment may be considered but clinicians are cautioned against unilateral treatment in cases with bilateral symptoms due to potential false negatives from corticosteroid use [63].

Intravitreal MTX is the most common ocular treatment [24, 116–119]. Standard injection doses are generally 400 µg/0.1 mL [120], though variations exist [121, 122]. No standardized protocol for injection frequency or number is established. Retrospective studies from 2008 to 2022 indicate that the most common complication is corneal epithelial damage (22.7%–100.0%), usually reversible with cessation or adjustment of treatment [121–126]. Other complications include conjunctival congestion, subconjunctival hemorrhage, cataracts, increased intraocular pressure, dry eye, vitreous hemorrhage, sterile endophthalmitis, optic nerve atrophy, macular disease, anterior segment ischemic syndrome, and endophthalmitis [122, 123, 127–130].

Rituximab, a chimeric monoclonal antibody targeting CD20-positive B cells, is used intravitreally at 1 mg/0.1 mL, administered monthly or weekly [131]. Side effects include transient increased intraocular pressure, granulomatous anterior uveitis, cataracts, and vitreous hemorrhage [132, 133]. Rituximab can reduce the number of MTX injections and related adverse effects despite its high cost.

Local radiation therapy, including both ocular and whole-brain radiation, was historically a first-line treatment for intraocular lymphoma [134, 135]. However, due to tumor recurrence, radiation-related ocular complications, and neuropsychiatric toxicity, ocular radiation is now less commonly used as a first-line treatment in recent years.

Retrospective studies show that intravitreal MTX (with or without systemic therapy) effectively alleviates ocular symptoms, with a complete response rate over 70% [123, 136]. Some studies report a 100% response rate after intravitreal MTX [22, 121, 122, 124, 126, 127, 129, 130, 136, 137]. Effectiveness is assessed through clinical presentation, imaging results, and intraocular fluid testing. Prospective and retrospective studies indicate that a decrease in aqueous humor IL-10 level is associated with treatment response [121, 138, 139]. Aqueous humor IL-10 level > 50 pg/mL is a viable threshold for detecting intraocular relapse (sensitivity 100% and specificity 95.1%), while the IL-10/IL-6 ratio is not related to the number of injections and is not an effective indicator of treatment response [76]. Recurrence can be managed with repeat MTX injections [123].

#### Clinical question 11: what are the recommendations for systemic treatment in patients with VRL?

Recommendation 24: Systemic treatment should be considered for VRL patients who can tolerate it and meet at least one of the following criteria: 1) Concurrent CNS and/or other systemic lymphomas; 2) Diagnosis of bilateral VRL; 3) CSF IL-10/IL-6 ratio > 1. (1b, A; Consensus level 96.67%).

Recommendation 25: Currently, there is no satisfactory systemic treatment regimen for VRL patients. Interdisciplinary collaboration such as hematology, neuro-oncology, radiotherapy, and other relevant departments is advised for the development of a treatment plan. The initial systemic treatment should be selected based on safety, cost-effectiveness, and patient compliance, and adjusted as needed based on changes in ocular and systemic conditions. (Expert Opinion; Consensus level 100%).

#### Basis of recommendation and evidence summary

Systemic treatment for VRL patients primarily includes systemic chemotherapy or immunochemotherapy, intrathecal chemotherapy, whole-brain radiotherapy, and autologous hematopoietic stem cell transplantation. Historically, due to limited understanding and low diagnosis rates of PVRL, patients often present with CNS or other systemic involvement. Systemic treatments typically followed protocols for PCNSL or other NHL. Systemic chemotherapy or immunochemotherapy is mainly based on high-dose methotrexate [136, 137, 140, 141], and may also include high-dose cytarabine [141, 142], ifosfamide [143, 144], or CHOP/R-CHOP (cyclophosphamide + doxorubicin + vincristine + prednisone, with or without rituximab) [117, 145, 146]. Intrathecal drugs mainly include MTX and/or cytarabine [137, 140, 145, 146]. Whole-brain radiotherapy (total dose 30–45 Gy) is primarily used for patients with CNS involvement [117, 128, 142, 145, 146], although some researchers have applied preventive reduced-dose whole-brain radiotherapy (total dose 23.4 Gy) for VRL patients without CNS involvement [142]. Autologous hematopoietic stem cell transplantation is used for recurrent or refractory patients, often with CNS involvement [147]. Recent advances in diagnostic techniques have led to more PVRL diagnoses, and high-intensity systemic treatments may cause more harm than benefit [146]. Consequently, safer and milder treatment options are being explored, including Bruton's tyrosine kinase inhibitors (ibrutinib, zanubrutinib, acalabrutinib) monotherapy [148–150], temozolomide monotherapy [151], and the R2 regimen (rituximab + lenalidomide induction, lenalidomide maintenance) [152, 153], with a beneficial result.

Several studies have compared the effects of local ocular treatment with or without systemic treatment and indicated that systemic treatment (mainly systemic chemotherapy or immunochemotherapy) can significantly delay CNS involvement in PVRL patients [123, 128, 140]. However, it does not significantly reduce intraocular or CNS relapse rates [123, 136]. The impact on progression-free survival and overall survival is still being debated [22, 117, 145, 146, 154].

For PVRL patients without systemic involvement, the 2011 International PCNSL Collaborative Group suggested a combination of ocular and systemic treatment for bilateral involvement [4]. The French Ocular Brain Lymphoma Network, established in 2011, recommended systemic treatment for patients in generally good condition and local ocular treatment for those in poorer condition [155]. Additionally, researchers have found CSF IL-10 to be a highly sensitive and specific biomarker in VRL patients [56]. Therefore, systemic treatment is recommended for PVRL patients with bilateral involvement and/or elevated CSF IL-10, provided their general condition allows for it.

Regarding treatment protocol, due to the low incidence and high clinical heterogeneity of the disease, current research on systemic treatment for VRL consists of retrospective studies or small sample, single-arm non-randomized controlled trials. There is no high-level evidence indicating the superiority of any particular treatment regimen. In summary, the systemic treatment plan for VRL patients should be developed collaboratively with hematology, neuro-oncology, radiotherapy, and other relevant departments, starting with the safest, most cost-effective, and patient-compliant regimen, and adjusted based on ongoing monitoring of ocular and systemic conditions.

#### Clinical questions on which consensus was not achieved

Due to variations in study design, methods, and populations across literature, the limitations of current published studies include small sample sizes, predominantly retrospective analyses, a lack of standardized diagnostic and treatment protocols, and diagnostic delays. Consequently, consensus could not be reached on the 12th clinical question (related to prognosis), which is not officially included in this guideline. However, given the significance of patient prognosis and follow-up in clinical practice, the relevant findings are presented here to guide future research in this area.

#### Clinical issue 12: what is the prognosis for patients with VRL?

Recommendation: Consensus not reached.

#### Basis of recommendation and evidence summary

There is substantial variation in clinical outcomes such as overall survival, progression-free survival, and CNS involvement reported across different studies. Overall survival for VRL patients is generally favorable, with a 5-year overall survival rate ranging from 68% [146] to 92.9% [136] in the absence of CNS involvement. However, when CNS involvement is present, the 5-year survival rate can decrease to 35% [146]. Patients presenting with both intraocular and CNS lesions at diagnosis have the shortest survival rate [117, 154]. Nearly half of PVRL patients may eventually develop CNS lymphoma [156], and current treatments do not fully prevent local recurrence or CNS involvement [22, 157].

Factors associated with a poor prognosis include: older age [26, 158], sub-RPE infiltrative lesions [23, 26, 159], serum IgA levels < 184 mg/dL [68], relatively high vitreous IL-35 levels [160], and *CD79B* Y196 gene mutations [161].

Regarding follow-up, the 2023 European Association of Neuro-Oncology (EANO) guidelines recommend at least fundoscopic and slit-lamp examinations, as well as brain MRI, for post-treatment follow-up of PCNSL patients [10]. The French LOC group suggests follow-up every six months for the first two years, and then annually [162]. Fundoscopic and slit-lamp examinations are non-invasive, cost-effective, and straightforward methods for routine follow-up. If patients have poor prognostic factors, additional imaging and intraocular fluid testing may be necessary based on the physician’s clinical judgment. Additionally, ophthalmologists should ensure regular brain MRI scans for early detection of CNS lesions. In 2019, the British Society for Hematology published guidelines for the diagnosis and management of primary CNS diffuse large B-cell lymphoma. Routine enhanced MRI is recommended during the course of treatment and monitoring. A suggested schedule is every 3 to 4 months for 2 years following completion of therapy and further surveillance MRI imaging should be judged on an individual patient basis [13].

Given the retrospective nature of existing evidence, limitations include small sample sizes, inconsistent treatment protocols, and significant variability in follow-up durations. Further high-quality randomized controlled trials with larger sample sizes are needed to validate the effectiveness of these potential prognostic markers. Moreover, individualized treatment and follow-up plans should be tailored to each patient’s specific situation.

## Conclusions

The working group systematically reviewed and comprehensively summarized the latest evidence from both domestic and international sources. Using the Oxford Evidence Level System, we assessed the quality of evidence and the strength of recommendations. Based on the practical experiences of clinical experts from various institutions and disciplines, and after thorough discussions over nine months, we developed the "Clinical Guidelines for the Diagnosis and Treatment of Vitreoretinal Lymphoma in Chinese Patients (2024)". This document provides crucial academic references and clinical practice guidance for the diagnosis and treatment of VRL patients. It includes VRL diagnostic methods, processes and treatment recommendations tailored to patients’ conditions and clinical realities, which are essential for the timely diagnosis and standardized treatment of VRL patients. Due to the rarity of VRL and the limited literature, especially the lack of randomized controlled trials, some recommendations in this guideline are based on lower levels of evidence or expert opinions. As VRL awareness and diagnosis and treatment technologies are continuously improving, more high-quality clinical research evidence will emerge, and the expert group will further update and refine the guidelines accordingly. Additionally, whether this guideline is useful for other ethic population is expected to be examined in the future study.

## Data Availability

Not applicable.

## Abbreviations

CSF: Cerebrospinal fluid
CNS: Central nervous system
DLBCL: Diffuse large B-cell lymphoma
EANO: European Association of Neuro-Oncology
FFA: Fluorescein fundus angiography
ICGA: Indocyanine green angiography
IgH: Immunoglobulin heavy chain
IL: Interleukin
MRI: Magnetic resonance imaging
MTX: Methotrexate
NHL: Non-Hodgkin’s lymphoma
OCT: Optical coherence tomography
OCTA: OCT angiography
PCNSL: Primary central nervous system lymphoma
PET: Positron emission tomography
PVRL: Primary vitreoretinal lymphoma
RPE: Retinal pigment epithelium
TCR: T-cell receptor
UWF: Ultra-wide field
VRL: Vitreoretinal lymphoma
